# Fermentation of *Panax notoginseng* root extract polysaccharides attenuates oxidative stress and promotes type I procollagen synthesis in human dermal fibroblast cells

**DOI:** 10.1186/s12906-020-03197-8

**Published:** 2021-01-14

**Authors:** Shiquan You, Xiuqin Shi, Dan Yu, Dan Zhao, Quan An, Dongdong Wang, Jiachan Zhang, Meng Li, Changtao Wang

**Affiliations:** 1 Beijing Advanced Innovation Center for Food Nutrition and Human Health, Beijing Technology and Business University, Fucheng Road, Beijing, 100048 China; 2 Chemistry and Materials Engineering, Beijing Technology and Business University, 11 Fucheng Road, Haidian District, Beijing, 100048 China; 3 Beijing Key Lab of Plant Resource Research and Development, Beijing Technology and Business University, Fucheng Road, Beijing, 100048 China; 4 Yunnan Baiyao Group Co., Ltd, Kunming, 650000 China

**Keywords:** *Panax notoginseng*, Polysaccharides, Fermentation, Oxidative stress, TGF-β/Smad signaling pathway

## Abstract

**Background:**

*Panax notoginseng* is one of the most valuable traditional Chinese medicines. Polysaccharides in *P. notoginseng* has been shown to significantly reduce the incidence of human diseases. However the application of fermentation technology in *Panax notoginseng* is not common, and the mechanism of action of *P. notoginseng* polysaccharides produced by fermentation is still unclear. The specific biological mechanisms of fermented *P. notoginseng* polysaccharides (FPNP) suppresses H_2_O_2_-induced apoptosis in human dermal fibroblast (HDF) and the underlying mechanism are not well understood.

**Methods:**

In this study, the effects of water extracted and fermentation on concentration of polysaccharides in *P. notoginseng* extracts were analyzed. After the H_2_O_2_-induced HDF model of oxidative damage was established, and then discussed by the expression of cell markers, including ROS, MDA, SOD, CAT, GSH-*Px* and MMP-1, COL-I, ELN, which were detected by related ELISA kits. The expression of TGF-β/Smad pathway markers were tested by qRT-PCR to determine whether FPNP exerted antioxidant activity through TGF-β signaling in HDF cells.

**Results:**

The polysaccharide content of *Panax notoginseng* increased after *Saccharomyces cerevisiae* CGMCC 17452 fermentation. In the FPNP treatment group, ROS and MDA contents were decreased, reversed the down-regulation of the antioxidant activity and expression of antioxidant enzyme (CAT, GSH-*Px* and SOD) induced by H_2_O_2_. Furthermore, the up-regulation in expression of TGF-β, Smad2/3 and the down-regulation in the expression of Smad7 in FPNP treated groups revealed that FPNP can inhibit H_2_O_2_-induced collagen and elastin injury by activating TGF-β/Smad signaling pathway.

**Conclusion:**

It was shown that FPNP could inhibit the damage of collagen and elastin induced by H_2_O_2_ by activating the TGF-β/Smad signaling pathway, thereby protecting against the oxidative damage induced by hydrogen peroxide. FPNP may be an effective attenuating healing agent that protects the skin from oxidative stress and wrinkles.

## Background

*Panax notoginseng* (Burk) F.H. Chen is a kind of Chinese herb with high medicinal value. The root of *Panax notoginseng* contains many chemical components such as saponins, flavonoids, volatile oils, amino acids, polysaccharides, various trace elements, etc. [1–3]. Relevant studies have shown that *P. notoginseng* has many pharmacological effects, and it has been used in a large proportion of clinical treatment of cardiovascular diseases [4], cerebrovascular [5], blood, nervous and immune systems [6], as well as wound healing [7–10]. It is also added as the main ingredient in topical Chinese patent medicines, such as Yunnan Baiyao and Pianzaihuang, for hemostasis, antiulcer and protection against premature aging. In these prescriptions, *P. notoginseng* was not added directly but hydrolyzed or fermented by microorganisms [11]. By adding concentrated *P. notoginseng* fermented extracts, these prescriptions can promote the synthesis of collagen to reduce fine lines and wrinkles and effectively repair damage caused by sunlight or the external environment [12].

The aqueous extracts of *P. notoginseng* are rich in polysaccharides [13, 14]. *P. notoginseng* polysaccharide (PNP) is a kind of heteroglycan which has been shown to have immunological [15], anticancer [16, 17], anti-hyperglycemic and anti-coagulation activity [18]. The PNP of high molecular weight is not conducive to absorption through the stratum corneum. To make PNP more efficient in protecting against the premature aging of the skin, low molecular weight PNP needs to be prepared. It has been reported that the conversion of macromolecular compounds into smaller forms using various microorganisms can improve their bioactivity [19, 20]. Fermentation is the process of using microbial enzymes or microorganisms to convert raw materials into desired products [11, 20, 21]. In our previous study*, P. notoginseng* strains fermented by various microorganisms were selected. *P. notoginseng* polysaccharides fermented by *Saccharomyces cerevisiae* CMCC17452 which isolated in yellow rice wine wheat starter showed the highest antioxidant activity [22]. The fermentation and extraction conditions of FPNP were isolated and optimization. This FPNP showed the activity of anti-inflammatory effects in the immortalized epidermal cell line HaCaT and anti-aging effects in HDF cells [23]. However, the relevance of these findings has yet to be fully documented, and the protective effect of FPNP on H_2_O_2_-induced oxidative stress and reduction of cellular collagen synthesis has not been studied.

Oxidative stress is a negative effect caused by the imbalance of oxidation and anti-oxidation in human body. Oxidative stress not only leads to increased protease secretion and production of a large number of oxidative intermediates, but also leads to increased collagen and elastin content in the dermis, further aging the skin [24]. However, antioxidant activity can be attributed to a variety of reactions and mechanisms, such as radical scavenging, reducing power, preventing chain initiation, etc. [25, 26]. Antioxidant effects should not only have the ability to clear reactive oxygen species (ROS) but also have the role of up-regulated related antioxidant enzymes, regulating redox cell signals and gene expression [27]. H_2_O_2_ is a kind of ROS produced in healthy cells during cellular respiration and metabolism. High concentrations of H_2_O_2_ may induce various human degenerative diseases and aging [28]. The cell viability and concentration of several markers of oxidative damage, including the generation of ROS and malondialdehyde (MDA), glutathione peroxidase (GSH-*Px*) the enzyme activity of catalase (CAT) and superoxide dismutase (SOD), are all affected by H_2_O_2_. Damage by H_2_O_2_ has been shown to affect the degradation of collagen and procollagen synthesis [29]. Therefore, in this study, HDF cells treated with hydrogen peroxide (H_2_O_2_) were used as a model to evaluate their antioxidant capacity. Transforming growth factor-β (TGF-β) is a pluripotent cytokine that that is reported to be involved in procollagen synthesis and has an irreplaceable role. H_2_O_2_ destroys the TGF-β/Smad pathway, further leading to reduction of type I procollagen [30] .

This study focused on detect the expression of oxidative damage markers and TGF-β/Smad gene in HDF cells, to determine whether FPNP regulates antioxidant enzymes through TGF-β signaling pathway, and then to study the mechanism of FPNP in HDF cells in antioxidant stress.

## Methods

### Reagents and instruments

Reagents and instruments were showed as following, including *Saccharomyces cerevisiae* CMCC17452, (isolated from yellow rice wine wheat in our laboratory); The *P.notoginseng* root (Wenshan in Yunnan Province, China); 2,2-diphenyl-1-picrylhydrazyl (DPPH) and ascorbic acid (Sigma-Aldrich,St. Louis, MO, USA); Fibroblast Medium (FM) (ScienCell, San Diego, CA, USA); Dulbecco’s Modified Eagle’s Medium (DMEM) and Fetal bovine serum (FBS), trypsin-EDTA and penicillin-streptomycin (Gibco Carlsbad, CA, USA); Cell lysis buffer for BCA Protein Assay Kit, Catalase (CAT), superoxide dismutase (SOD), Malondialdehyde (MDA) and ROS kits (Shanghai Beyotime Biotechnology Shanghai, China). Huma COL-1 (Collagen 1) ELISA Kit, Human Matrix metalloproteinase-1 (MMP-1) ELISA Kit and Huma Elastin (ELN) ELISA Kit (CUSABIO,Wuhan, China); EasyScript® One-Step gDNA Removal and cDNA Synthesis SuperMix and TransStart® Top Green qPCR SuperMix (TransGen Biotech Beijing, China). All other chemicals used were of high purity biochemistry grade. Olympus Inverted fluorescent microscope (Shanghai Tulsen Vision Technology Co., LTD,Shanghai, China); Infinite M200 PRO fluorescence marker (Deken Trading Co., LTD).

### Fermentation of RGE

*S. cerevisiae* CMCC17452 was cultivated with YPD broth (Becton, Dickinson and Company, Sparks, MD, USA) at 28 °C for 48 h. *P. notoginseng* root (PNR) and pure water were were mixed to a ratio of 1 to 10 before sterilization for 15 min at 121 °C. *S. cerevisiae* CMCC17452 was inoculated into PNR solution at a concentration of 5% (V/V) and fermented at 28 °C for 24 h. The PNR solution without *S. cerevisiae* CMCC17452 was used as a blank control. Samples were centrifuged and the supernatant collected for analysis and named fermented *P. notoginseng* root liquid (FPNR) and water-extracted *P. notoginseng* root liquid (WPNR).

### Measurement of polysaccharide, ginsenoside and flavonoid content

Polysaccharide content was measured using the sulfuric acid method [31]. The entire saponin content was determined using the modified vanillin-acetic acid assay with a standard curve of ginsenoside [32]. The entire flavonoid content was measured using a colorimetric assay with rutin as the standard sample.

### Measurement of antioxidant activity in vitro

DPPH, hydroxy free and superoxide anion free radical scavenging activity were performed following the published methods [10, 33].

### Cell culture and MTT assay

Human dermal fibroblast cells (HDF, from Shanghai Institutes for Biological Sciences) were grown in FM medium containing 2% FBS, 1% fibroblast growth additive and 1% penicillin/streptomycin at 37 °C in 5% CO_2_ atmosphere. We change culture medium every two days and split using 0.25% trypsin and 0.02% EDTA solution at 80 to 90% confluency.

Cells were seeded at 1× 10^5^ cells/mL in a 96-well plate for 12 h and incubated with various concentration of FPNP (0.25–2.5 mg·mL^− 1^) for 12 h. Next, the cells were incubated with a 10 μL MTT solution (100 mmol·L^− 1^) for 4 h. Subsequently, 100 μL of dimethyl sulfoxide (DMSO, Sigma) was added to dissolve the purple formazan crystals. The optical densities of the solutions were quantified at a wavelength of 595 nm using a infinite M200 PRO fluorescence marker.

Cell survival is the percentage of viable cells compared to the control sample in the presence of the extract. Both untreated cells and DMEM cells of the same volume were used as controls, and each sample passed three independent analytical tests [34].

### Establishment of oxidative stress model

The cells were inoculated on a 96-well plates at a concentration of 8× 10^4^ for oxidative stress analysis. When establishing the oxidative damage model of HDF caused by H_2_O_2_, according to cell viability, the IC_50_ concentration and time of the cells treated by H_2_O_2_ were determined using SPSS software. The concentrations of H_2_O_2_ were set at 1, 10, 25, 50, 100, 250, 500 and 1000 μmol·L^− 1^.

### Determination of intracellular ROS, MDA, SOD, CAT and GSH-Px

Superoxide dismutase (SOD) was assayed by the method as references [35]. The assay is based on total Superoxide Dismutase Assay Kit with WST-8 in line, relies on WST-8 [2-(2-methoxy-4-nitrophenyl)-3-(4-nitro- phenyl)-5-(2,4-disulfophenyl)-2H-tetrazolium], which produces a highly water-soluble formazan dye, the reaction inhibited by SOD. Reactive Oxygen Species (ROS) level was detected by the method as references [35]. The assay is based on 2′,7′ -dichlorofluorescin-diacetate (DCFH-DA), which can be oxidized into fluorescent DCF; The activity of catalase (CAT) was determined by the method as references [36], the catalase Assay Kit (Beyotime China) based on the protocols provided by the manufacturer. Glutathione peroxidase (GSH-Px) was estimated by the method as reference [37]. This is commercially available kit(Beyotime, China) according to the manufacturer’s instructions.Malondialdehyde(MDA ) was estimated by the method as reference [38], this is lipid peroxidation MDA assay kit (Beyotime, China) based on the thiobarbituric acid.

### Measurement of MMP-1, COL-I and ELN production

Cells were seeded at 1× 10^7^ (cells/well) in a 6 well plate for 8–12 h, then incubate with or without FPNP for 24 h, furthermore treated with H_2_O_2_ to oxidative stress, HDF cells in 6-well plates were cultured again with FM from ScienCell (San Diego, CA, USA). After 24 h, the supernatant was collected and the COL-I, MMP-1 and ELN content were measured using a corresponding assay ELISA kit purchased from Cusabio (Wuhan, China) (in strict accordance with the kit manufacturer’s protocols) for anti-aging analysis.

### Quantitative reverse transcriptional PCR

Cells were seeded at 1× 10^7^ (cells/well) in a 6 well plate for 8–12 h, then incubate with or without FPNPt for 24 h, furthermore treated with H_2_O_2_ to oxidative stress. Trizol was used to extract the total RNA from the cells according to the instructions, then the EasyScript® One-Step gDNA Removal and cDNA Synthesis SuperMix was used for cDNA synthesized according to the manufacturer’s instructions. qPCRs were performed with TransStart® Top Green qPCR SuperMix. Each reaction was performed in three replicate samples. The internal control gene is the GADPH gene. The relative expression levels were calculated using 2 -ΔΔCt. The primer sequence list is based on the study of Jihong Wu et al [39]. 

### Statistical analysis

Three separate experiments were conducted for all the experiments, each sample underwent three technical repeats and analyses. The mean standard deviation is going to be the results. One factor Analysis of Variance (ANOVA) and Dunnett test were used to analyze the data to determine which pairs were significantly different. Statistical analyses were conducted using GraphPad Prism7 (GraphPad Software, Inc., La Jolla, CA, USA). *p*< 0.05 was considered significant.

## Results

### Fermentation of PNR by *Saccharomyces cerevisiae* CGMCC 17452

The concentrations of polysaccharides, ginsenosides and flavonoids were measured in water-extracted PNR (WPNR) and fermented PNR (FPNR) by *S. cerevisiae* CGMCC 17452. As can be seen from Table 1, there are significant differences between WPNR and FPNR in polysaccharides and ginsenosides, and during fermentation, the content of both is increased. The content of water-soluble flavonoids did not rise in FPNR. Compared to WPNR, FPNR had higher levels of polysaccharide, which may be because *S. cerevisiae* CGMCC 17452 strains plays an important role in the extraction of PNR.

**Table 1:** Concentration of polysaccharides, ginsenosides and flavonoids in WPNR

Concentration (μg/ml)	WPNR	FPNR
Polysaccharides	39.8±2.02^a^	41.28±0.32^b^
Ginsenosides	0.44±0.06^c^	0.84±0.05^d^
Flavonoids	6.14±1.44^e^	5.98±0.79^e^

Values are presented as mean standard deviation a-e Means in the same column followed by different letters represent significant differences by concentration (*p* < 0.05)

WPNR: water-extracted *P. notoginseng* root

FPNR: *P. notoginseng* root fermented with *Saccharomyces cerevisiae*

### Extraction of WPNP and FPNP, and measurement of antioxidant activity

The polysaccharides of WPNR and FPNR were extracted from the hot water extraction solution and fermentation respectively by alcohol precipitation, then DEAE-Sepharose Fast Flow anion exchange chromatography was used to purify and named “WPNP” and “FPNP”. Three components of the polysaccharides were isolated and purified. The structure and compositions of purified polysaccharide were analyzed by infrared spectral and gel permeation chromatography respectively in our previous study [23].

DPPH radical scavenging activity, superoxide anion radical scavenging activity and hydroxyl radical scavenging activity were used to determine antioxidant activity. According to the three used assays, FPNP exhibited statistically significant higher antioxidant activity in comparison to WPNP (Table 2).

**Table 2:** Antioxidant effects of WPNP and FPNP

Sample	Concentration (mg/ml)	DPPH radical scavenging activity (%)	Hydroxy free radical scavenging activity (%)	Superoxide anion free radical scavenging activity (%)
WPNP	0.5	13.82±0.12^ab^	33.45±0.47^a^	56.48±1.16^a^
	1	15.16±0.69^ab^	68.78±0.93^b^	63.71±0.70^b^
	2	51.71±0.44^c^	75.54±0.35^cd^	66.42±2.49^c^
	2.5	57.27±0.48	79.03±0.62^cd^	68.39±2.06^d^
	5	76.14±1.20^d^	96.55±0.06^e^	76.35±0.65^e^
FPNP	0.5	17.22±0.34^a^	31.51±0.81^a^	68.28±1.54^ab^
	1	40.79±067^b^	63.64±1.19^b^	69.54±0.62^ab^
	2	49.24±0.61^c^	76.92±0.36^c^	72.21±0.42^cd^
	2.5	57.13±0.16^d^	90.19±0.10^d^	72.49±1.53^cd^
	5	62.77±0.71^e^	95.45±0.34^e^	75.09±0.27^e^

Values are presented as mean standard deviation a-e Means in the same column followed by different letters represent significant differences by concentration (*p* < 0.05)

### Effects of FPNP on H2O2 treated in HDF cells

**Fig. 1:**
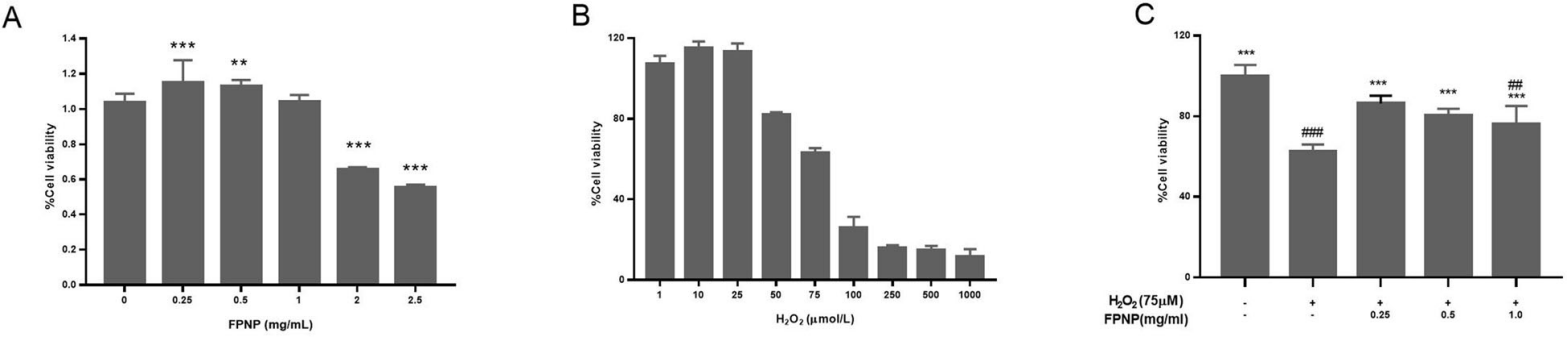
Effects of FPNP on H_2_O_2_-induced cytotoxicity in HDF cells. (**a**) Effects of FPNP on the viability of HDF cells. (**b**) Effects of HDF survival rate caused by H_2_O_2_. (**c**) FPNP prevents H_2_O_2_-induced cytotoxicity in HDF cells. The data is presented as the mean ± SD of the six experiments in each group. Number signs indicate a highly significant difference from the control cells. ##: *p*< 0.01; ###: *p*< 0.001. Asterisks indicate a highly significant difference from the H_2_O_2_-treated cells. *: *p*< 0.05, **: *p*< 0.01

The cytotoxic character of FPNP and H_2_O_2_-induced toxicity was examined by MTT assay. When the concentration of FPNP reached 1.0 mg·mL^− 1^, cell proliferation activity still exceeded 100%, indicating that FPNP has no toxicity to cells. When the FPNP concentration was 0.5 mg·mL^− 1^, the cell survival rate was 112.8%, seeing Fig. 1a. The results show that the low concentration of FPNP solution had a proliferation effect and an effect on promote the proliferation of HDF cells.

For further confirmation, we also tested the effect of FPNP on H_2_O_2_-induced cytotoxicity. Figure 1b shows the cell survival rate when different concentrations of H_2_O_2_ were used to stimulate HDF for 2 h. With the increase in H_2_O_2_ concentration, the HDF survival rate gradually decreases. Finally, the oxidative damage model of HDF was established by treating HDF with 75 μmol·L^− 1^ H_2_O_2_ as the optimum dose of this study.

As shown in Fig. 1c, H_2_O_2_-treated HDF cells showed a decreased survival rate, while treatment by FPNP cells showed a dose-dependent recovery of reduced cell activity. 0.25 mg·mL^− 1^ of FPNP recovered viability by 38.96% compared with H_2_O_2_-damaged cells. The viabilities of cells treated with H_2_O_2_ and FPNP (0.25, 0.5 and 1.0 mg·L^− 1^) showed statistical difference (*p*< 0.01), and the three levels of 0.25, 0.5 and 1.0 mg·mL^− 1^ of treatment concentration were selected for further study. These results suggest that FPNP can protect HDF cells from H_2_O_2_-induced cell viability reduction.

### Effects of FPNP on H2O2-induced ROS generation, MDA production and ABTS scavenging activities in HDF cells

**Fig. 2:**
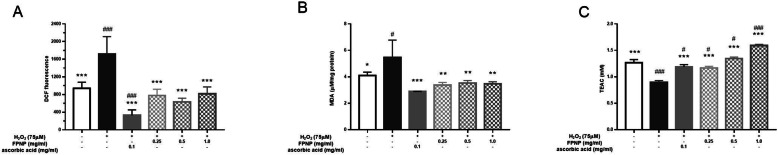
(**a**) Intracellular ROS scavenging capacities of different concentrations of FPNP of H_2_O_2_-induced oxidative stress in HDF cells. (**b**) Effects of FPNP on MDA content in H_2_O_2_-induced oxidative stress in HDF cells. (**c**) Effects of FPNP on total antioxidant capacity in H_2_O_2_-induced oxidative stress in HDF cells. All data is shown as the mean ± SD of at least three independent experiments. Number signs indicate a highly significant difference from the control cells. ##: *p*< 0.01; ###: *p*< 0.001. Asterisks indicate a highly significant difference from the H_2_O_2_-treated cells. *: *p*< 0.05, **: *p*< 0.01

The statistics of the experimental data are shown in Fig. 2a, with the stimulation of H_2_O_2_, ROS levels were significantly augmented. The positive control was ascorbic acid when evaluating the antioxidant effect of FPNP. After treatment with 0.1 mg·mL^− 1^ of ascorbic acid, the ROS level significantly decreased than H_2_O_2_-treated group (*p*< 0.01). FPNP also markedly reduced the levels of intracellular ROS (*p*< 0.01), compared with the H_2_O_2_-treated model group. When H_2_O_2_-induced cells were treated with 0.5 mg·mL^− 1^ of FPNP, the ROS level was reduced by 57.29%. These results show that FPNP can protect cells from H_2_O_2_ oxidative damage by reducing the ROS level.

With the stimulation of H_2_O_2_, the effects of FPNP on MDA in cells are shown in Fig. 2b and total antioxidant capacity is shown in Fig. 2c. The positive control was ascorbic acid and was statistically analyzed (*p*< 0.01). In H_2_O_2_-induced HDF cells, 0.25, 0.5 and 1.0 mg·mL^− 1^ FPNP decreased the MDA content (*p*< 0.01) and increased the total antioxidant capacity of the cells (*p*< 0.05). These results show that FPNP can protect cells from H_2_O_2_-induced damage by reducing their MDA content and increasing their total antioxidant capacity.

### Effects of FPNP on CAT, GSH-Px and SOD activity in H2O2-induced HDF cells

**Fig. 3:**
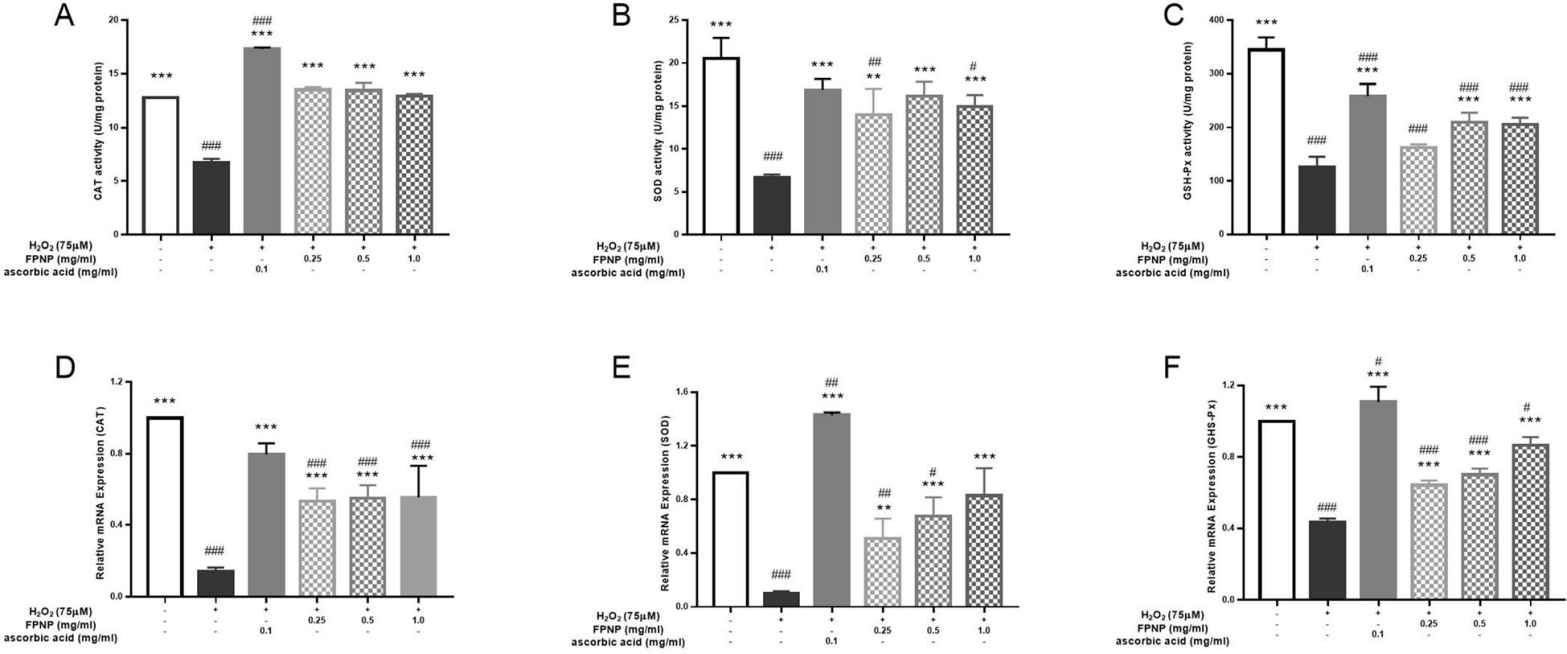
Effects of FPNP on H_2_O_2_-induced changes in cellular antioxidant enzymes CAT (**a**), SOD (**b**) and GSH-*Px* (**c**) in HDF cells. The mRNA expression of H_2_O_2_-induced oxidative stress metabolism-related genes includes CAT (**d**), SOD (**e**) and GSH-Px (**f**) in HDF cells pretreated with different concentrations of FPNP. Each mRNA is normalized to ribosomal protein GAPDH and expressed relative to the control level. All data is shown as the mean ± SD of at least three independent experiments. Number signs indicate a highly significant difference from the control cells. ##: *p*< 0.01; ###: *p*< 0.001. Asterisks indicate a highly significant difference from the H_2_O_2_-treated cells. *: *p*< 0.05, **: *p*< 0.01

The activity of antioxidant enzymes in cells were measured, including CAT, GSH-*Px* and SOD. See Figs. 3, 0.25, 0.50, and 1.00 mg·mL^− 1^ FPNP increased CAT, GSH-*Px* and SOD activity compared with the H_2_O_2_-treated model group. As can be seen from Fig. 3a, CAT activity was significantly increased in FPNP group, compared with model group (*p*< 0.01), with a significant difference, and was consistent with that of the control group (*p*> 0.05). SOD activity was highest, when the concentration of FPNP was 0.50 mg/ml. the difference between the H_2_O_2_ treatment model group and the control group was statistically significant (*p*< 0.01). Under these conditions, the effects are similar to those of 0.10 mg·mL^− 1^ of ascorbic acid, as shown in Fig. 3b. When the concentrations of FPNP were 0.50 mg·mL^− 1^ and 1 mg·mL^− 1^, the level of GSH-*Px* activity was increased significantly compared with model group (p< 0.01), as shown in Fig. 3c.

0.25, 0.50, and 1.00 mg·mL^− 1^ of FPNP significantly increased the expression of CAT, GSH-*Px* and SOD relative to mRNA expression. The expression of the CAT-expression mRNA level was similar in the FPNP treatment group at different concentrations and markedly increased in the H_2_O_2_-treated group (*p*< 0.01), compared with the model group. as shown in Fig. 3d. There was an apparent dose-dependent relationship between the expression level of SOD-related mRNA and FPNP dosage: as the concentration of FPNP increased, the expression level increased. As can be seen from Fig. 3e, it was similar to the control group, with no significant difference, when the concentration of FPNP was 1.00 mg·mL^− 1^.The expression of GSH-*Px* mRNA in the 1.00 mg·mL^− 1^ FPNP treatment group was the highest, and there was little difference from that of the control group, as shown in Fig. 3f. In summary, these results clearly show that FPNP can repair oxidative stress damage by increasing the activity of CAT, GSH-*Px* and SOD, and the expression of related mRNA in HDF cells.

### Effects of FPNP on H2O2-induced MMP-1, col-I and ELN contents

**Fig. 4:**
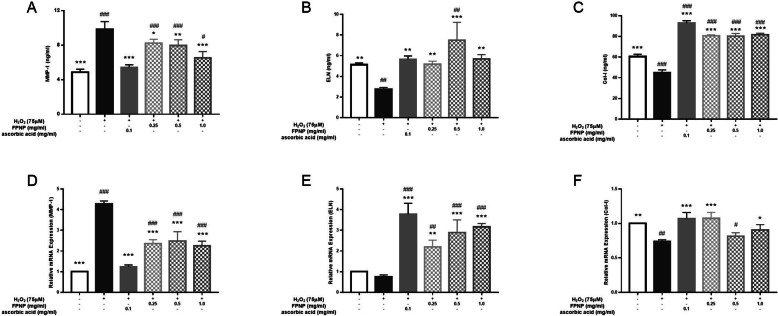
Effects of different concentrations of FPNP on H_2_O_2_-induced changes in an intracellular reduced ratio of MMP-1(**a**), ELN (**b**) and COL-I (**c**) in HDF cells. The mRNA expression of related genes includes MMP-1 (**d**), ELN (**e**) and COL-I (**f**) in HDF cells pretreated with different concentrations of FPNP. Each mRNA is normalized to ribosomal protein GAPDH and expressed relative to the control level. Number signs indicate a highly significant difference from the control cells. ##: *p*< 0.01; ###: *p*< 0.001. Asterisks indicate a highly significant difference from the H_2_O_2_-treated cells. *: *p*< 0.05, **: *p*< 0.01

H_2_O_2_ affects collagen and elastin synthesis by stimulating MMP-1. As shown in Fig. 4a, 1.00 mg·mL^− 1^ of FPNP significantly reduced the content of MMP-1 in the culture medium of H_2_O_2_-induced HDF cells (*p*< 0.01) and achieved anti-aging effects. Effect of different concentrations of FPNP on secretions of ELN and Col-I as shown in Fig. 4b and Fig. 4c, FPNP can significantly increase secretions of ELN and Col-I in H_2_O_2_-induced HDF cell cultures. There was no significant difference between 0.50 mg·mL^− 1^ FPNP and 0.10 mg·mL^− 1^ ascorbic acid, while 0.50 mg·mL^− 1^ of FPNP significantly improved the contents of ELN and Col-I.

The results of qRT-PCR show that Compared with the control group, MMP-1 mRNA expression in HDF cells was significantly increased (*p*< 0.01), and the expression of Col-I and ELN mRNA was inhibited. After FPNP treatment, the expression of MMP-1 mRNA decreased, indicating that 0.25, 0.50, and 1.00 mg·mL^− 1^ of FPNP can inhibit the upregulation of MMP-1 mRNA expression in the H_2_O_2_-treated model group (*p*< 0.01), seeing Fig. 4d. With FPNP treatment, both ELN and Col-I mRNA expression increased over the H_2_O_2_-treated model group and came close to those of the control level (*p*< 0.01; Fig. 4e and f). This indicates that FPNP can effectively improve the secretion of ELN and Col-I and achieve anti-aging effects.

### TGF-β/Smad pathway signaling in HDF cells

**Fig. 5:**
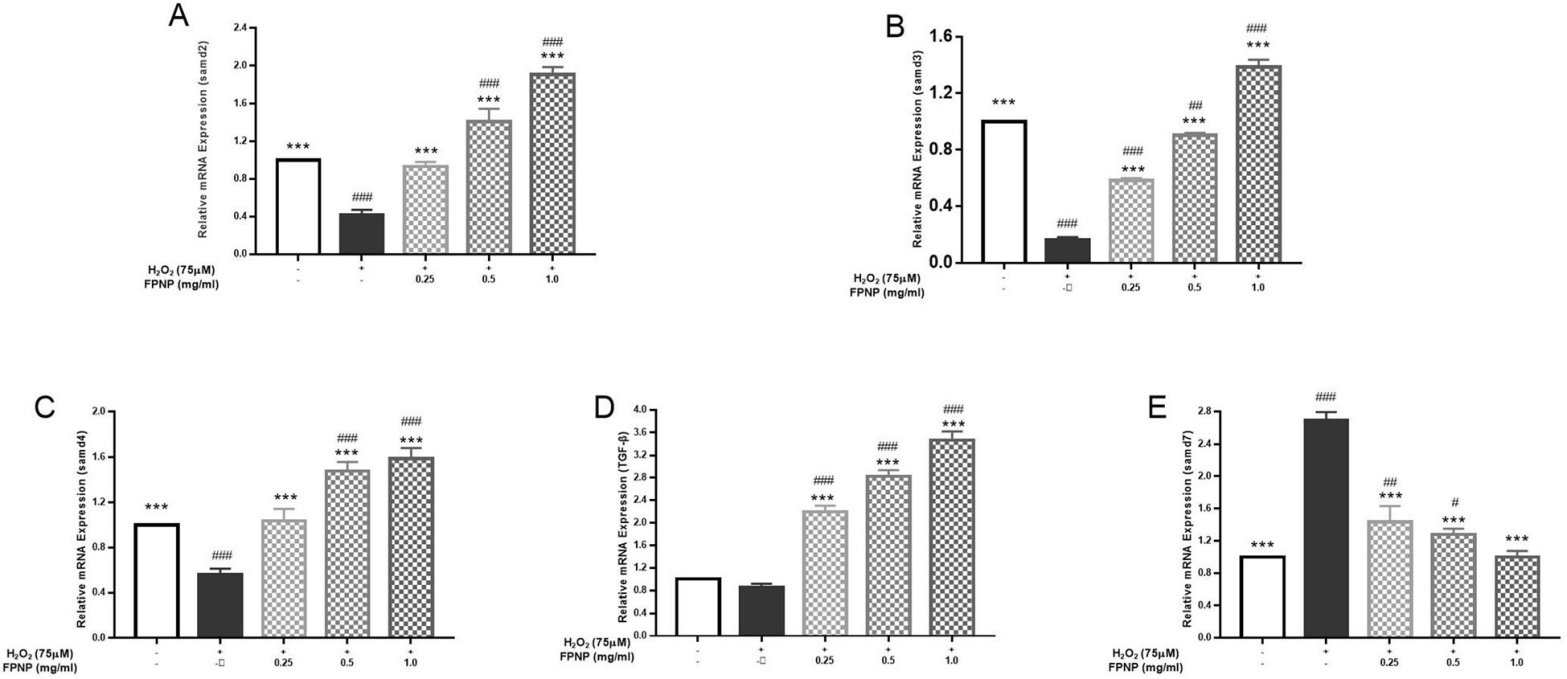
Effects of different concentrations of FPNP on H_2_O_2_-induced changes of mRNA expression of TGF-β/Smad signaling pathway-related genes including Smad2(**a**), Smad3(**b**), Smad4(**c**), TGF-β(**d**) and Smad7(**b**) in HDFs cells pretreated with different concentrations of FPNP, and each mRNA is normalized to ribosomal protein GAPDH and expressed relative to the control level

Transforming growth factor-β (TGF-β) has been recognized as a strong mediator in the synthesis of collagen and elastin. To clarify the effects of FPNP on Col-I and ELN synthesis, the expression of TGF-Smad1, Smad2, Smad3 and Smad7 mRNA in HDF induced by H_2_O_2_ were detect by qRT-PCR. Seeing in Fig. 5, the results of qRT-PCR showed that compared with those of the control group, H_2_O_2_ significantly downregulated the expressions of TGF-β1 and Smad 2/3 (*p*< 0.01), while FPNP reversed this effect. 1.00 mg·mL^− 1^ of FPNP recovered the expression of *smad2*, *smad3* and *smad4* mRNA expression 3.58-fold, 7.53-fold and 1.84-fold respectively compared with the H_2_O_2_ group, and increased TGF-β1 mRNA 3.04-fold compared with the H_2_O_2_ group, as shown in Fig. 5a-d. In addition, we investigated the effect of FPNP on the expression of *smad7*, which a negative factor in the TGF-β/Smad pathway. Seeing Fig. 5e, 1.00 mg·mL^− 1^ of FPNP downregulated the Smad7 mRNA expression level by 62.84% compared with the H_2_O_2_ group. This experimental evidence suggests that FPNP leads to the overexpression of TGF-β in HDF, which in turn inhibits H_2_O_2_-induced collagen and elastin injury.

## Discussion

*P. notoginseng* polysaccharide (PNP) is a kind of heteroglycan which has been shown to have immunological [15], anticancer [16, 17], anti-hyperglycemic and anti-coagulation activity [18]. It has been reported that the conversion of macromolecular compounds into smaller forms using various microorganisms can improve their bioactivity [19, 20]. With reference to the lactic acid bacteria fermentation of *Panax notoginseng*, HPLC analyses showed that the fermentation increased the content of *Panax notoginsenosides* and other active substances reported in previous studies [40], in our study, the fermented *P. notoginseng* polysaccharide (FPNP) were prepared by *S. cerevisiae*. The results showed that fermentation increased the polysaccharide content of *Panax notoginseng*, which was in accordance with literature reports. HDF cells treated with hydrogen peroxide were used as a model to simulate the oxidative stress response. We used HDF cells treated with hydrogen peroxide as a model to simulate the oxidative stress response, so as to study the mechanism of antioxidant stress injury caused by the fermentation of *Panax notoginseng* polysaccharides.

In skin aging, histological and ultrasonic structural studies have shown that enhanced epidermal thickness and changes in connective tissue are common features of damaged skin [41–44]. Collagen and elastin are the proteins responsible for the strength and elasticity of the skin. Ultraviolet radiation triggers the activation of MMPs, which attack and degrade collagen and elastin, leading to photoaging of the skin. Inhibition of MMPs is considered an effective strategy to prevent UV-induced photoaging. Oxidative stress could induce matrix metalloproteinase-1 (MMP-1) [45], In this study, hydrogen peroxide accelerated the production of collagen-soluble MMP-1 in cells. MMP-1 degrades collagen, which are the main structural proteins of the dermal extracellular matrix (ECM), and maintain the strength and elasticity of the skin [46]. We found FPNP (1.00 mg·mL^− 1^) treatment can reduce the production of MMP-1. FPNP can reduce the loss of collagen and elastin in cells. On this basis, FPNP not only inhibits the expression of MMP-1, but also promotes the expression of structurally related proteins in extracellular matrix to exert its protective effect. Our results support that FPNP can be used to prepare cosmetics to repair and regenerate essential proteins in photo-aging skin. Column chromatography was used to isolate *Panax notoginseng* root, and two new polysaccharides, MAP and MRP5A, have antioxidant and anti-aging effects, reported in previous studies [47]. In subsequent studies, the structure of FPNP can be separated to study the specific components that play a role.

Smad protein is the downstream transmembrane receptor of TGF-β and is an important regulatory molecule of TGF-β superfamily signaling [48]. Oxidative stress can induce MMPs and pro-inflammatory cytokines in cells. TGF-β promotes the synthesis of collagen type I [41]. Therefore, preparing anti-inflammatory compounds for inflammation in advance seems to be a strategy to improve skin oxidative stress. Hydrogen peroxide promotes the secretion of Smad7, which subsides after FPNP treatment. At the same time, FPNP promotes the expression of TGF-β1, Smad2, Smad3, Smad4. It is possible that FPNP elevates type I collagen levels in part by enhancing the TGF-β/Smad pathway. Previous studies [45] have demonstrated that UVB induced lipid peroxidation, apoptosis and MMP-1 expression in fibroblasts are similar to our results, indicating that oxidative stress damage caused by hydrogen peroxide is similar to that caused by UVB.

**Fig. 6:**
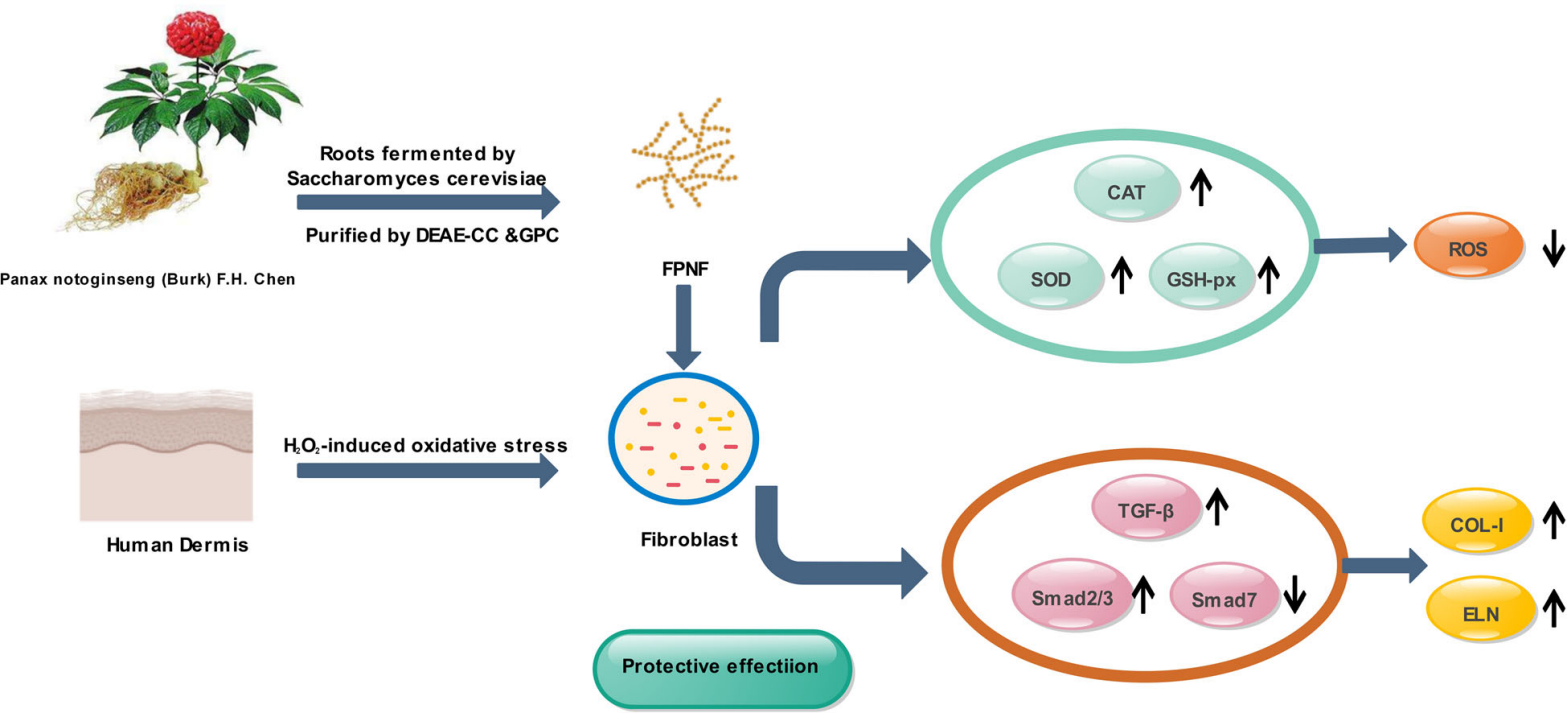
Scheme summarizing the inhibition of H_2_O_2_-induced oxidant injury by FPNP via the up-regulation of antioxidant enzymes and TGF-β/Smad pathway

It is reported that ROS is related to the production of MMP and the breakdown of collagen [43]. Therefore, controlling ROS levels seems to be a major mechanism through which plant compounds with antioxidant activity can be particularly effective. The ethanol extract from Dalbergia bug protects skin keratinocytes from oxidative stress by inhibiting the production of reactive oxygen species. Topical application of patchouli alcohol inhibit UV-induced aging of mouse skin caused by reactive oxygen species [49]. In our study, as shown in Fig. 6, FPNP cleared ROS, which explained that in HDF cells, increased SOD, CAT and GSH-*Px* levels. These protective properties of FPNP seem to be accomplished by up-regulating the expression of cellular antioxidant genes.

## Conclusion

We have provided evidence that FPNP represses H_2_O_2_-induced HDF fibroblast injury, including the elevation of ROS and MDA content, decreased expression of antioxidant-related enzymes (CAT, GSH-*Px* and SOD), increased MMP-1 content and reduced collagen and elastin. Moreover, FPNP efficiently H_2_O_2_-mediates oxidative reparation, such as the elevation of collagen and elastin content through the TGF-β/Smad signaling pathway. Ultimately, we propose that FPNP may be an effective attenuated healing agent for protecting the skin against oxidative stress and wrinkles.

## Data Availability

The data used and/or investigated during the present study are available from the corresponding author upon reasonable request. t.

## Abbreviations

FPNP: Fermented *P. notoginseng* polysaccharides
FPNR: Fermented *P. notoginseng* root liquid
WPNR: Water-extracted *P. notoginseng* root liquid
HDF: Inhuman dermal fibroblast
PNP: *P. notoginseng* polysaccharide
PNR: *P. notoginseng* root
ROS: Reactive oxygen species
MDA: Malondialdehyde
GSH-*Px*: Lutathione peroxidase
CAT: Catalase
SOD: Superoxide dismutase
TGF-β: Transforming growth factor-β
DPPH: 2,2-diphenyl-1-picrylhydrazyl
FM: Fibroblast Medium
DMEM: Dulbecco’s Modified Eagle’s Medium
FBS: Fetal bovine serum
MMP-1: Matrix metalloproteinase-1
H2-DCFDA: 2′, 7′ -dichlorodihydrofluorescein diacetate
